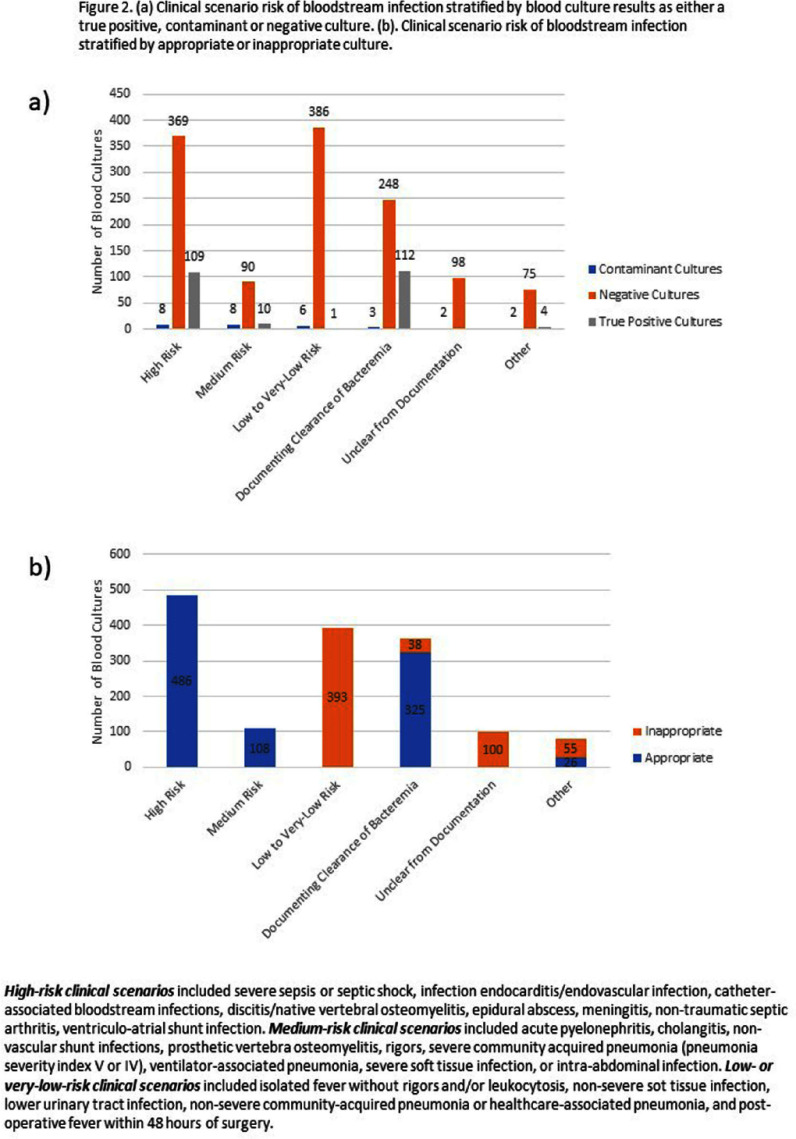# VAD or Bad? Implementing a Blood Culture Algorithm in Ventricular Assist Device Patients

**DOI:** 10.1017/ash.2025.307

**Published:** 2025-09-24

**Authors:** Jessica Seidelman, Erin Gettler, Heather Pena, Lynn McGugan, Becky Smith, Deverick Anderson, Patrick Tam

**Affiliations:** 1 Duke University; 2 Duke University Medical Center; 3 Duke University Health System; 4 Duke University Medical Center; 5 Duke Center for Antimicrobial Stewardship and Infection Prevention

## Abstract

**Introduction:** Blood culture (BCx) diagnostic stewardship is crucial for optimizing health resources and ensuring appropriate clinical testing while minimizing unnecessary cultures that could lead to increased false positives and subsequent antibiotic overuse. BCx algorithms have effectively lowered BCx rates across various patient populations without compromising patient safety. However, patients with a durable left ventricular assist device (LVAD) represent a unique group where the safety and applicability of these algorithms remain underexplored. **Methods:** We adapted the BCx algorithm from the DISTRIBUTE study by Fabre et al (Figure 1) and retrospectively applied it to HeartMate 3 LVAD recipients with BCx testing performed between July 1, 2019, and April 30, 2024. Each BCx was reviewed and adjudicated according to the algorithm to determine the appropriateness of BCx indication. We also assessed the incidence of true positives, contaminants, and negative cultures among BCx testing deemed as inappropriate to evaluate the algorithm’s potential impact on clinical decision-making in this specialized patient population. We used the Centers for Disease Control and Prevention’s standard definition of a contaminated BCx. **Results:** We reviewed 1531 blood cultures in 121 unique LVAD recipients. The most common clinical indications for BCx collection were for documenting bloodstream clearance (363, 23.7%), suspected infective endocarditis or endovascular infection (260, 17.0%), and isolated fever and/or leukocytosis (217, 14.2%). We adjudicated 945 (61.7%) BCx collections as appropriate and 586 (38.3%) as inappropriate. Out of the 586 inappropriate BCx collections, 577 (98.5%) were negative and 8 (1.4%) resulted in a contaminant (Figure 2). Only 1 (0.2%) BCx adjudicated as inappropriate resulted in a true positive, which isolated Streptococcus infantarius in an LVAD patient receiving active chemotherapy for colorectal cancer and was felt to represent gastrointestinal translocation. **Discussion:** We retrospectively applied a BCx algorithm to LVAD recipients to determine the clinical impact of applying such an algorithm to a high-risk patient population. We found that the BCx algorithm missed only 1 true positive bloodstream infection in a patient with additional risk factors. This study provides preliminary support that a BCx algorithm could reduce BCx testing in LVAD recipients without compromising clinical safety. Future studies on BCx diagnostic stewardship in this population should prospectively collect data and monitor for additional adverse events, such as readmission, mortality, length of stay, and antibiotic days of therapy.

**Figure:**
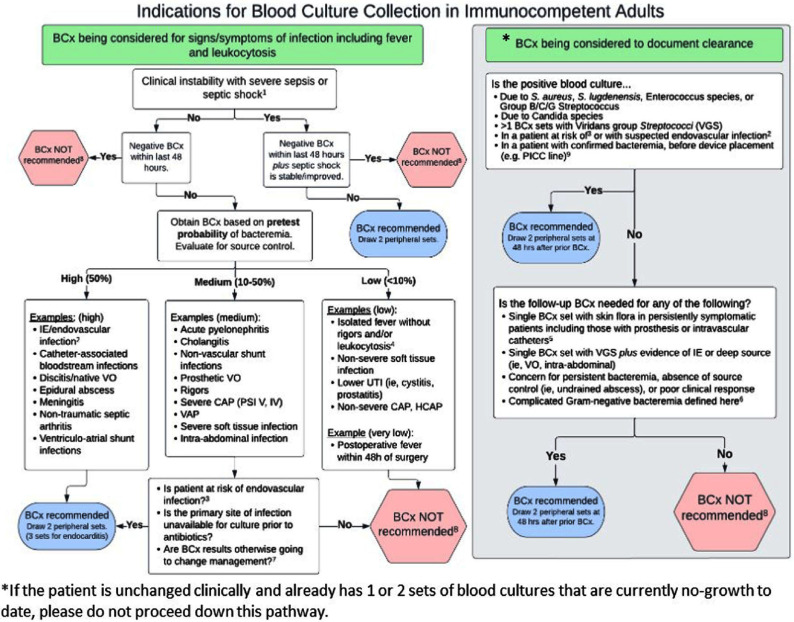

**Figure:**